# The Beta Agonist Lung Injury TrIal (BALTI) - prevention trial protocol

**DOI:** 10.1186/1745-6215-12-79

**Published:** 2011-03-15

**Authors:** Gavin D Perkins, Daniel Park, Derek Alderson, Matthew W Cooke, Fang Gao, Simon Gates, Sarah E Lamb, Dipesh Mistry, David R Thickett

**Affiliations:** 1 University of Warwick, Warwick Medical School Clinical Trials Unit, Warwick, CV4 7AL, UK; 2 Department of Surgery, University of Birmingham, Birmingham, B15 2TT, UK; 3 University of Birmingham, Birmingham, B15 2TT, UK

## Abstract

**Background:**

Acute lung injury complicates approximately 25-30% of subjects undergoing oesophagectomy. Experimental studies suggest that treatment with beta agonists may prevent the development of acute lung injury by decreasing inflammatory cell infiltration, activation and inflammatory cytokine release, enhancing basal alveolar fluid clearance and improving alveolar capillary barrier function.

**Methods/Design:**

The Beta Agonist Lung Injury TrIal (prevention) is a multi-centre, randomised, double blind, placebo-controlled trial. The aim of the trial is to determine in patients undergoing elective transthoracic oesphagectomy, if treatment with inhaled salmeterol 100 mcg twice daily started at induction of anaesthesia and continued for 72 hours thereafter compared to placebo affect the incidence of early acute lung injury and other clinical, resource and patient focused outcomes. The primary outcome will be the development of acute lung injury within 72 hours of oesophagectomy. The trial secondary outcomes are the development of acute lung injury during the first 28 days post operatively; PaO_2_: FiO_2 _ratio; the number of ventilator and organ failure free days, 28 and 90 day survival; health related quality of life and resource utilisation. The study aims to recruit 360 patients from 10 UK centres.

**Trial registration number:**

Current Controlled Trials ISRCTN47481946

## Background

Acute Lung Injury (ALI) and the Acute Respiratory Distress Syndrome (ARDS) occur in response to a wide variety of insults including traumatic injuries, sepsis, multiple blood transfusions, pneumonia or aspiration of gastric contents. The syndrome carries with it significant morbidity[1], a significant risk of death[2], prolonged hospital stay and high treatment costs[3]. The pathophysiological changes seen in ALI/ARDS comprise systemic and alveolar inflammation, increased pulmonary vascular permeability and alveolar capillary damage[4]. This causes flooding of the alveolar space which reduces pulmonary compliance, interferes with ventilation perfusion matching and results in hypoxaemia, and acute respiratory failure.

ALI/ARDS complicates approximately 25-30% of subjects undergoing oesophagectomy[5-8]. One lung ventilation which is used during the transthoracic approach to oesophagectomy is implicated in the pathogenesis of ALI/ARDS[4,9]. It invokes an ischaemia-reperfusion injury on the deflated lung, whilst the ventilated lung is exposed to oxygen toxicity and high inflation pressures which cause ventilator associated lung injury. Thus patients undergoing oesophagectomy with one lung ventilation are at high risk of developing ALI/ARDS and may serve as a group of people in whom prophylactic strategies to prevent the development of lung injury may be considered[10].

### Rationale for beta agonists in the prevention of ALI/ARDS

#### Inflammation

β_2 _agonists reduce neutrophil sequestration, activation and inflammatory cytokine production *in-vitro *and in animal models of ARDS[11]. In humans, inhaled salmeterol (long acting β_2 _agonist) given prior to lipopolysaccharide inhalation reduces neutrophil influx, degranulation and tumour necrosis factor α release[12]. However in the single centre randomised controlled trial (BALTI-1), although salbutamol increased circulating neutrophil numbers, it had no effect on alveolar neutrophil sequestration, activation or alveolar cytokine levels[13]. A potential explanation for this finding is that the administration of a β_2 _agonist after the clinical manifestations of ARDS are apparent may be too late to prevent the early inflammatory influx.

#### Endothelial / epithelial function

The balance between oedema formation and clearance is influenced by the degree of alveolar-capillary permeability, pulmonary capillary hydrostatic and oncotic pressures, and rate of active alveolar fluid clearance[14]. β_2 _agonists may reduce oedema formation through enhancing alveolar-capillary barrier function. Animal and human studies [15,16] report improvement in endothelial barrier function as well as providing epithelial cytoprotection against infection[11]. In BALTI-1, there was *in-vivo *evidence of reduced alveolar capillary permeability and *in-vitro *evidence of enhanced epithelial monolayer wound repair in subjects treated with salbutamol[17].

Experimental studies in animals, and *ex-vivo *human lung show that both intravenous and inhaled β adrenergic agonists accelerate the rate of alveolar fluid clearance thus hastening resolution of non-cardiogenic pulmonary oedema[18]. Evidence of their potential efficacy in humans was first shown by Sartori *et al *who used inhaled salmeterol to prevent the development of high altitude pulmonary oedema in mountaineers[19]. In patients with ARDS, the phase II randomised controlled trial BALTI-1 found that intravenous salbutamol significantly reduced lung water and improved lung mechanics ARDS[20].

In summary, the potential of prophylactic administration of β_2 _agonists in subjects at high risk of developing ARDS has not previously been evaluated. Theoretically the administration of treatment prior to injury has the potential to reduce the development of lung injury through decreasing inflammatory cell infiltration, activation and inflammatory cytokine release, enhancing basal alveolar fluid clearance and improving alveolar capillary barrier function. This study aims to define the effectiveness of inhaled salmeterol (a long acting β_2 _agonist) for the prevention of ALI/ARDS in a group of subjects at high risk of developing the condition. The treatment is simple and known to be well tolerated in people with obstructive lung diseases and if effective could be easily incorporated into widespread clinical practice. The results from this trial will provide the basis for future clinical studies investigating this and other preventative strategies in broader subject groups known to be at risk of ARDS (such as pneumonia and sepsis).

The Beta Agonist Lung Injury TrIal (prevention) [BALTI-prevention] is a multi-centre, randomised, double blind, placebo-controlled trial. The aim of the trial is to determine in patients undergoing elective transthoracic oesphagectomy, if treatment with inhaled salmeterol 10 0mcg twice daily started at induction of anaesthesia and continued for 72 hours thereafter compared to placebo affect the incidence of early acute lung injury and other clinical, resource and patient focused outcomes.

## Methods / Design

### Trial Approvals and Conduct

The trial is approved by South Birmingham Research Ethics committee. The trial is registered on the International Standard Randomised Controlled Trial Registry (ISRCTN47481946). The sponsor organisation for the trial is Heart of England NHS Foundation Trust. The trial is being coordinated by the Warwick Clinical Trials Unit (http://www.warwick.ac.uk/go/ctu). The trial is funded by the National Institute for Health Research (NIHR) Research for Patient Benefit Programme (PB-PG-0408-16104). Further details can be found on the trial website (http://www2.warwick.ac.uk/fac/med/research/ctu/trials/ecr/baltiprevention). The trial will be carried out in accordance with the Medical Research Council (MRC) Good Clinical Practice Guidelines, applicable UK legislation and the Standard Operating Procedures of the Warwick Clinical Trials Unit. The trial will be reported in line with the Consolidated Standards of Reporting Trials (CONSORT) 2010 guidelines[21].

### Outcome measures

#### Primary outcome

The primary outcome will be the development of acute lung injury within 72 hours of oesophagectomy. Lung injury will be defined by the American European Consensus conference definition[22] as the acute onset of (1) Bilateral infiltrates on the chest xray (2) Hypoxaemia with a PaO_2 _: FiO_2 _ratio of < 40 kPa (3) absence of clinical evidence of left atrial hypertension. The presence of absence of ALI will be determined by a trial end-point committee who will review chest xrays and physiological data from trial participants. The time window of 72 hours has been selected to exclude ALI/ARDS which may occur as a consequence of surgical complications e.g. anastamotic leak, ventilator associated pneumonia which occur later in the post-operative course.

#### Secondary outcomes

The trial secondary outcomes are the development of ALI/ARDS during the first 28 days post operatively; PaO_2 _: FiO_2 _ratio; the number of ventilator and organ failure free days, 28 and 90 day survival; health related quality of life and resource utilisation.

Ventilator free days are defined in accordance with the ARDSnet criteria[23] as the number of calendar days after initiating unassisted breathing to day 28 after randomisation, assuming a patient survives for at least 48 consecutive hours after initiating unassisted breathing. Un-assisted breathing is defined as one of at least 48 consecutive hours of (1) being extubated with face mask, nasal prong oxygen, or room air (2) T-tube breathing (3)Tracheostomy mask breathing, CPAP = 5 cm H_2_0 without pressure support of intermittent mandatory ventilation assistance. For example, if a subject initiates unassisted breathing on day 16 and survives to day 28, he/she will be assigned a value of 12 VFDs. If a similar subject begins unassisted breathing on day 16 but dies on day 25, the VFDs is 9. If a subject survives for >48 consecutive hours of unassisted breathing but requires assisted breathing (for any reason) before day 28, he/she will be assigned only the number of days of unassisted breathing before day 28. Subjects who die without initiating unassisted breathing or before 48 consecutive hours of unassisted breathing will be assigned a value of zero VFDs. Subjects transferred to another hospital or other health care facility prior to day 28 (intermediate care, nursing home etc.) while still on positive pressure ventilation will be followed up to assess this efficacy measure.

Organ failure free days are defined in a similar manner to ventilator free days with an organ failure free day being a day without evidence of non-respiratory organ failure. Organ failure will be defined as a sequential organ failure assessment (SOFA) score of greater than 3[24].

Health related quality of life will be measured using EuroQol (EQ-5D). Data will be collected at baseline, 28 days and 90 days. Resource utilisation (intensive care and hospital length of stay) will be recording. The cost of intervention and resource use will be compared.

#### Translational sub-study

A translational sub-study will run in a subset of patients enrolled in the main trial. In addition to the outcomes above extravascular lung water (PiCCO, Pulsion Medical) will be measured and bronchoalveoalar lavage fluid[25,26] and plasma will be collected to measure markers of inflammation, tissue damage / repair.

### Eligibility criteria

Patients will be eligible for the trial if they fulfil the following criteria:

1. Provision of informed consent

2. Age >18 years

3. Planned elective transthoracic oesophagectomy

4. Ability to use inhaler with spacer device

Patients fulfilling any of the criteria below will be excluded from the trial:

1. Pregnancy

2. Current treatment with long acting beta agonist (any formulation)

3. Allergy to excipients contained in the salmeterol inhaler (HFA 134a)

4. Current treatment with non-cardioselective beta blockers (the cardioselective beta blockers atenolol, betazolol, bisoprolol and metoprolol are permitted).

5. Treatment with an investigational medicinal product in the last 30 days.

### Power and sample size estimate

A systematic review of the literature noted significant variability in the incidence of acute lung injury after oesophagectomy with reports ranging from 14.5 - 43%[5]. Major studies relevant to the UK population include the national CEPOD report which quotes an incidence of 27%[8] and national gastro-oesophageal audit(ASCOTT) which had an incidence 40.5%[7]. An analysis of 6971 patients from Intensive Care National Audit and Research Centre (ICNARC) found that 3352 (47%) fulfilled the oxygenation criteria for ALI in the first 24 hours post operatively[6]. A 5 year audit of oesophagectomies at Heart of England NHS Trust identified that 28% of patients fulfilled criteria for ALI. Based on these data a conservative estimate of the incidence ALI of 25% was selected.

There are no prospective randomised controlled trials in oesophagectomy patients to predict the size of the treatment effect of salmeterol for preventing ALI. In the randomised controlled high altitude pulmonary oedema study, the prophylactic use of salmeterol reduced the incidence of pulmonary oedema from 74 to 33% (55% relative reduction)[13]. LPS challenge studies in humans have shown that salmeterol can reduce neutrophil influx, inflammatory cytokine production and aberrant coagulation by 40-66% relative reduction[12,27]. The study originally set out to demonstrate a 15% absolute reduction in development of acute lung injury. However the study funders requested in 2009 that the study was re-powered to detect a more modest treatment effect. The size of the treatment effect was decided following discussions with the British Society of Oesophageal Gastric Surgeons. Assuming a baseline incidence of lung injury of 25%, consensus was reached that a treatment effect of at least 12.5% (50% relative reduction) was necessary to convince clinicians to translate the treatment into clinical practise should the trial demonstrate that salmeterol prevents ALI/ARDS. This equates to a number need to treat of eight to prevent one episode of lung injury.

**Figure 1:**
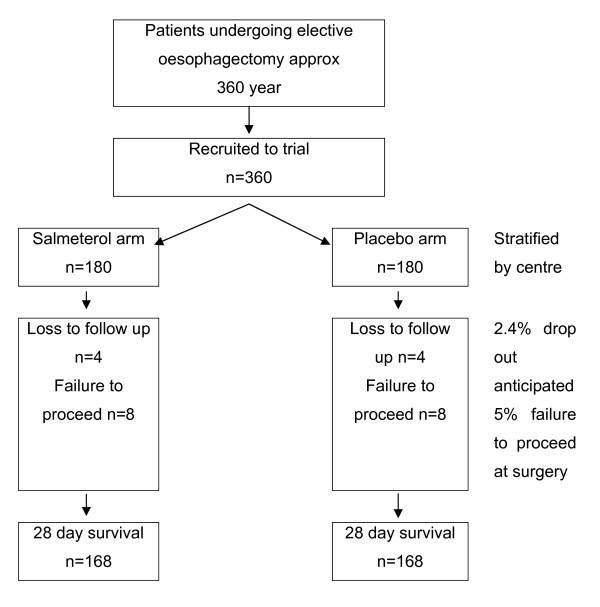
**Trial flow diagram**.

To demonstrate this effect with 80% power at a significance level of 0.05 will require 336 patients. To ensure the study maintains sufficient power after drop-outs we have included a 5% failure to proceed to oesphagectomy / receive one lung ventilation and a 2.4% loss to follow-up rate[28]. This gives an overall sample size of 180 per arm (figure 1). This sample size will have 80% power to detect the following relative risk changes in secondary outcomes at a significance level of 0.05: PaO_2 _: FiO_2 _ratio (10%); critical care (35%) and hospital (15%) length of stay; HRQL (15%) and breathlessness(20%).

### Trial conduct

Approach to patients and obtaining informed consent

Patients will be identified through upper GI cancer teams. Eligible patients will be invited to participate by their treating clinician, specialist clinical nurse or research nurse. If agreeable, written informed consent will be obtained, following a face to face discussion about the study.

#### Randomisation and drug / placebo supply

The trial statistician will produce the randomisation sequence using a block size of 10 with equal allocation between active and placebo groups. Drug (salmeterol 25 mcg metered dose inhaler) and matching placebo will be supplied and packaged according to the randomisation sequence into numbered treatment boxes by MODEPHARMA(London, UK). Drug boxes will be supplied in groups of ten to centres thus ensuring an equal allocation between active and placebo groups to balance any differences in case mix, pre-operative, operative and post-operative care between centres. Patients will be randomised sequentially by allocating them to the next numbered treatment pack held at the centre.

#### Drug administration

Subjects will receive either 100 mcg inhaled salmeterol or placebo via a spacer device within two hours of induction of anaesthesia and then 12 hourly for 72 hours. The drug will be administered by qualified medical or nursing staff. Whenever possible, subjects will be extubated at the end of surgery. Subjects requiring ventilation post operatively will receive 100 mcg of the drug via an in-line chamber inserted into the inspiratory limb of the ventilator circuit.

#### Concomitant medications

The following medications should not be prescribed to patients for the duration of the clinical study. Long acting beta agonists, non-cardioselective beta blockers (the cardioselective beta blockers atenolol, betazolol, bisoprolol and metoprolol are permitted) or another investigational medicinal product.

Inhaled or nebulised short acting beta agonists should be avoided whenever possible, but are permitted in this trial in the event of severe bronchospasm.

#### Post randomisation withdrawals and exclusions

Subjects may withdraw from the trial or the trial treatment at any time without prejudice. If a subject withdraws from the trial treatment, then they will be followed-up wherever possible and data collected as per protocol until the end of the trial. The only exception to this is where the subject also explicitly withdraws consent for follow-up.

#### Protocol compliance

Compliance with study drug administration will be recorded in the data collection booklet by the trial team by cross referencing with the subjects individual trial drug pack and drug treatment chart.

#### Blinding / un-blinding

Patients, clinical and research / trial staff will be unaware of which arm of the study a patient is allocated. Active and placebo treatment packs and their contents will be identical in appearance. The protocol allows for emergency un-blinding in the event of significant concerns about patient safety. In the unlikely event that un-blinding is required the local investigator will discuss with the Chief Investigator. All events will be logged.

#### Monitoring and reporting adverse events

Balti-prevention is recruiting a population who are prone to recognized medical and surgical complications. It is expected that many of the patients will experience an event that might be seen as a serious adverse event but is a recognized complication following oesophagectomy. Adverse and serious adverse events which are recognised complications of surgery e.g. medical (pneumonia, sepsis) and surgical (chyle leak, anastamosis leak) will be recorded in the case report form.

Death, other serious adverse events thought to be related to the study drug or serious unexpected serious adverse reactions will be reported to the trial co-ordinating centre and Chief Investigator within 24 hours of becoming aware of their occurrence who will inform the sponsor and regulatory authorities.

#### Data collection

Data up until hospital discharge will be recorded in each subject's Case Report Form (CRF) by hospital staff. Most of the data collected will be obtained from the patients hospital notes. In the unlikely event that a subject is transferred to another hospital, the study team will ensure that data collection is completed by the receiving hospital.

If the subject remains in hospital at 28 or 90 days, survival at these time points will be recorded by hospital staff. Mortality after hospital discharge will be obtained from NHS Statistical Tracing Service (NSTS).

Health related quality of life follow-up at 28 days and 90 days will use face to face interview if the subject is still in hospital or postal questionnaire. Any subject who has moved house without informing the trial team will be traced via the NHS Strategic Tracing Service. The questionnaire will contain the EuroQoL (EQ-5D) and resource use questions.

Postal questionnaires will be sent out and collected by WCTU. Questionnaires that are not returned, will be chased using a standard protocol involving reminders by post (2), telephone (2) and if necessary, collection of core data items by telephone.

### Statistical analysis plan

The primary analysis will be based on 'Intention-to-treat' (ITT). In the ITT analyses, patients will be analysed according to the treatment they were randomised to, irrespective of the treatment they actually received. In addition a per-protocol analysis will be conducted. Patients that did not proceed with planned surgery or did not receive the pre-operative dose of drug will be excluded.

Multiple imputation methods will be used to impute missing data. Prior to an imputation, the data mechanisms (MAR - missing at random; NMAR - not missing at random; MCAR - missing completely at random) will be assessed to make sure that multiple imputation is viable. Only data that is validly missing will be imputed. In the case of multivariate normal data, the multiple imputation methods assuming normality will be used. In the case where one cannot assume a distribution of the data, the ICE (imputation by chain equations) will be used. The imputed (intention to treat and adherence to protocol) datasets will be used for sensitivity analyses.

The primary outcome measure will be compared between treatment groups using a logistic regression model, with the dependent variable as acute lung injury/no acute lung injury within 72 hours and the independent variables as treatment and other important predictors (e.g. centre and age). An odds ratio measuring the treatment effect and its 95% confidence interval will be reported.

Categorical data will be analysed using logistic regression models, with treatment group as an independent variable along with other important predictors. The summary statistics will be based on proportions and the 95% CI. Continuous data: Continuous data will be analysed using linear regression models, with treatment group as an independent variable along with other important predictors. Difference in treatment will be based on adjusted mean estimates and 95% CI's. Time to event data will be analysed using a log-rank test. Any patients who have not experienced an event at the time point of interest or withdrawn will be censored. The proportion experiencing an event over time will be illustrated using a Kaplan-Meier curve for each of the treatment groups. The p-values and a hazard ratio with its 95% CI from a Cox proportional hazards model will also be presented. The proportional hazard assumption across treatment arms will be checked graphically using a log-cumulative hazard plot.

### Trial organisation / oversight

Trial oversight will be provided by a Trial Steering Committee (TSC) comprising of investigators, clinicians and trialists. The TSC will operate within the relevant WCTU SOP. An independent data monitoring committee will monitor the safety of participants enrolled in the trial through regular review of adverse event reports. An interim analysis of efficacy is not planned.

## Abbreviations

AE: Adverse event; ALI: Acute lung injury; ARDS: Acute respiratory distress syndrome; BAL: Bronchoalveolar lavage; CRF: Case Report Form; CTU: Clinical Trials Unit; DMEC: Data Monitoring and Ethics Committee; HAPE: High Altitude Pulmonary oedema; ICNARC: Intensive Care National Audit and Research Centre; NSTS: NHS Strategic Tracing Service; OLV: One lung ventilation; SAE: Serious adverse event; SUSAR: Suspected unexpected serious adverse reaction; TSC: Trial Steering Committee; VFD: Ventilator free days; WCTU: Warwick Clinical Trials Unit.

## Competing interests

The placebo inhalers for this trial were provided through an unrestricted educational grant from GlaxoSmithKline. GDP, DRT and FG received a research grant from GlaxoSmithKline to investigate the effects of salmeterol on alveolar inflammation in ARDS. GDP and DRT have received lecture fees and reimbursement of expenses from GlaxoSmithKline.

## Authors' contributions

All authors made a substantial contribution to the protocol development. All authors have approved this manuscript.

## References

[B1] Dowdy DW Eid MP Dennison CR Mendez-Tellez PA Herridge MS Guallar E Pronovost PJ Needham DM Quality of life after acute respiratory distress syndrome: a meta-analysis Intensive Care Med 2006 32 8 1115 1124 10.1007/s00134-006-0217-3 16783553

[B2] Brun-Buisson C Minelli C Bertolini G Brazzi L Pimentel J Lewandowski K Bion J Romand JA Villar J Thorsteinsson A Epidemiology and outcome of acute lung injury in European intensive care units. Results from the ALIVE study Intensive Care Med 2004 30 1 51 61 10.1007/s00134-003-2022-6 14569423

[B3] Cheung AM Tansey CM Tomlinson G az-Granados N Matte A Barr A Mehta S Mazer CD Guest CB Stewart TE Two-year outcomes, health care use, and costs of survivors of acute respiratory distress syndrome AmJ RespirCrit Care Med 2006 174 5 538 544 10.1164/rccm.200505-693OC 16763220

[B4] Baudouin SV Lung injury after thoracotomy BrJAnaesth 2003 91 1 132 142 10.1093/bja/aeg083 12821572

[B5] Park D Goureveith D Perkins GD Vincent JL Lung injury after oesophagectomy Yearbook of Intensive Care and Emergency Medicine Volume 29 2008 Germany: Springer 155 160

[B6] Park D Welch C Harrison D Palser T Cromwell D Gao F Alderson D Rowan K Perkins G Outcomes following oesophagectomy in patients with oesophageal cancer: a secondary analysis of the ICNARC Case Mix Programme Database Critical Care 2009 13 Suppl 2 S1 10.1186/cc7868 20003248 PMC2791299

[B7] McCulloch P Ward J Tekkis PP Mortality and morbidity in gastro-oesophageal cancer surgery: initial results of ASCOT multicentre prospective cohort study BMJ 2003 327 7425 1192 1197 10.1136/bmj.327.7425.1192 14630753 PMC274052

[B8] Sherry K Gray AJG, Hoile RW, Ingram GS, Sherry K Management of patients undergoing oesophagectomy Report of the National Confidential Enquiry Into Peri-Operative Deaths 1997 London: National CEPOD 57 61

[B9] Schilling MK Gassmann N Sigurdsson GH Regli B Stoupis C Furrer M Signer C Redaelli C Buchler MW Role of thromboxane and leukotriene B4 in patients with acute respiratory distress syndrome after oesophagectomy BrJAnaesth 1998 80 1 36 40 10.1093/bja/80.1.36 9505775

[B10] Ware LB Matthay MA The acute respiratory distress syndrome NEnglJMed 2000 342 18 1334 1349 10.1056/NEJM200005043421806 10793167

[B11] Perkins GD McAuley DF Richter A Thickett DR Gao F Bench-to-bedside review: beta2-Agonists and the acute respiratory distress syndrome Crit Care 2004 8 1 25 32 10.1186/cc2417 14975042 PMC420065

[B12] Maris NA de Vos AF Dessing MC Spek CA Lutter R Jansen HM van der Zee JS Bresser P van der Poll T Antiinflammatory Effects of Salmeterol after Inhalation of Lipopolysaccharide by Healthy Volunteers American Journal of Respiratory and Critical Care Medicine 2005 172 7 878 884 10.1164/rccm.200503-451OC 15994467

[B13] Perkins GD Nathani N McAuley DF Gao F Thickett DR In vitro and in vivo effects of salbutamol on neutrophil function in acute lung injury Thorax 2007 62 1 36 42 10.1136/thx.2006.059410 16928710 PMC2111273

[B14] Berthiaume Y Matthay MA Alveolar edema fluid clearance and acute lung injury Respir Physiol Neurobiol 2007 159 3 350 359 10.1016/j.resp.2007.05.010 17604701 PMC2682357

[B15] McAuley DF Frank JA Fang X Matthay MA Clinically relevant concentrations of beta2-adrenergic agonists stimulate maximal cyclic adenosine monophosphate-dependent airspace fluid clearance and decrease pulmonary edema in experimental acid-induced lung injury Crit Care Med 2004 32 7 1470 1476 10.1097/01.CCM.0000129489.34416.0E 15241090

[B16] Basran GS Hardy JG Woo SP Ramasubramanian R Byrne AJ Beta-2-adrenoceptor agonists as inhibitors of lung vascular permeability to radiolabelled transferrin in the adult respiratory distress syndrome in man EurJ NuclMed 1986 12 8 381 384 10.1007/BF00252194 2878809

[B17] Perkins GD Gao F Thickett DR In vivo and in vitro effects of salbutamol on alveolar epithelial repair in acute lung injury Thorax 2008 63 3 215 220 10.1136/thx.2007.080382 17951278

[B18] Matthay MA Folkesson HG Clerici C Lung epithelial fluid transport and the resolution of pulmonary edema Physiol Rev 2002 82 3 569 600 12087129 10.1152/physrev.00003.2002

[B19] Sartori C Allemann Y Duplain H Lepori M Egli M Lipp E Hutter D Turini P Hugli O Cook S Salmeterol for the prevention of high-altitude pulmonary edema NEnglJ Med 2002 346 21 1631 1636 10.1056/NEJMoa013183 12023995

[B20] Perkins GD McAuley DF Thickett DR Gao F The beta-agonist lung injury trial (BALTI): a randomized placebo-controlled clinical trial AmJRespirCrit Care Med 2006 173 3 281 287 10.1164/rccm.200508-1302OC 16254268

[B21] Schulz KF Altman DG Moher D CONSORT 2010 Statement: updated guidelines for reporting parallel group randomised trials Trials 2010 11 32 10.1186/1745-6215-11-32 21350618 PMC3043330

[B22] Bernard GR Artigas A Brigham KL Carlet J Falke K Hudson L Lamy M LeGall JR Morris A Spragg R Report of the American-European Consensus conference on acute respiratory distress syndrome: definitions, mechanisms, relevant outcomes, and clinical trial coordination. Consensus Committee JCrit Care 1994 9 1 72 81 10.1016/0883-9441(94)90033-7 8199655

[B23] Assessment of Low tidal Volume and elevated End-expiratory volume to Obviate Lung Injury (ALVEOLI): Trial protocol http://www.ardsnet.org/system/files/alveoli_protocol_and_amendments_0.pdf 10.1177/08850666211028139 34165010

[B24] Ferreira FL Bota DP Bross A Melot C Vincent JL Serial evaluation of the SOFA score to predict outcome in critically ill patients JAMA: The Journal of the American Medical Association 2001 286 14 1754 1758 10.1001/jama.286.14.1754 11594901

[B25] Perkins GD Chatterjee S Giles S McAuley DF Quinton S Thickett DR Gao F Safety and tolerability of nonbronchoscopic lavage in ARDS Chest 2005 127 4 1358 1363 10.1378/chest.127.4.1358 15821216

[B26] Perkins GD Chatterjie S McAuley DF Gao F Thickett DR Role of nonbronchoscopic lavage for investigating alveolar inflammation and permeability in acute respiratory distress syndrome Crit Care Med 2006 34 1 57 64 10.1097/01.CCM.0000190197.69945.C5 16374157

[B27] Maris NA de Vos AF Bresser P van der Zee JS Jansen HM Levi M van der PT Salmeterol enhances pulmonary fibrinolysis in healthy volunteers Crit Care Med 2007 35 1 57 63 10.1097/01.CCM.0000249827.29387.4E 17080003

[B28] Harvey S Harrison DA Singer M Ashcroft J Jones CM Elbourne D Brampton W Williams D Young D Rowan K Assessment of the clinical effectiveness of pulmonary artery catheters in management of patients in intensive care (PAC-Man): a randomised controlled trial Lancet 2005 366 9484 472 477 10.1016/S0140-6736(05)67061-4 16084255

